# Inhibitor of the Nuclear Transport Protein XPO1 Enhances the Anticancer Efficacy of KRAS G12C Inhibitors in Preclinical Models of KRAS G12C–Mutant Cancers

**DOI:** 10.1158/2767-9764.CRC-21-0176

**Published:** 2022-05-10

**Authors:** Husain Yar Khan, Misako Nagasaka, Yiwei Li, Amro Aboukameel, Md. Hafiz Uddin, Rachel Sexton, Sahar Bannoura, Yousef Mzannar, Mohammed Najeeb Al-Hallak, Steve Kim, Rafic Beydoun, Yosef Landesman, Hirva Mamdani, Dipesh Uprety, Philip A. Philip, Ramzi M. Mohammad, Anthony F. Shields, Asfar S. Azmi

**Affiliations:** 1 Barbara Ann Karmanos Cancer Institute, Department of Oncology, Wayne State University School of Medicine, Detroit, Michigan.; 2 University of California Irvine School of Medicine and Chao Family Comprehensive Cancer Center, Orange, California.; 3 Division of Neurology, Department of Internal Medicine, St. Marianna University, Kawasaki, Japan.; 4 Karyopharm Therapeutics, Newton, Massachusetts.

## Abstract

The identification of molecules that can bind covalently to KRAS G12C and lock it in an inactive GDP-bound conformation has opened the door to targeting KRAS G12C selectively. These agents have shown promise in preclinical tumor models and clinical trials. FDA has recently granted approval to sotorasib for KRAS G12C–mutated non–small cell lung cancer (NSCLC). However, patients receiving these agents as monotherapy generally develop drug resistance over time. This necessitates the development of multi-targeted approaches that can potentially sensitize tumors to KRAS inhibitors. We generated KRAS G12C inhibitor–resistant cell lines and observed that they exhibit sensitivity toward selinexor, a selective inhibitor of nuclear export protein exportin 1 (XPO1), as a single agent. KRAS G12C inhibitors in combination with selinexor suppressed the proliferation of KRAS G12C–mutant cancer cell lines in a synergistic manner. Moreover, combined treatment of selinexor with KRAS G12C inhibitors resulted in enhanced spheroid disintegration, reduction in the number and size of colonies formed by G12C-mutant cancer cells. Mechanistically, the combination of selinexor with KRAS G12C inhibitors suppressed cell growth signaling and downregulated the expression of cell-cycle markers, KRAS and NF-κB as well as increased nuclear accumulation of tumor suppressor protein Rb. In an *in vivo* KRAS G12C cell-derived xenograft model, oral administration of a combination of selinexor and sotorasib was demonstrated to reduce tumor burden and enhance survival. In conclusion, we have shown that the nuclear transport protein XPO1 inhibitor can enhance the anticancer activity of KRAS G12C inhibitors in preclinical cancer models.

**Significance:**

In this study, combining nuclear transport inhibitor selinexor with KRAS G12C inhibitors has resulted in potent antitumor effects in preclinical cancer models. This can be an effective combination therapy for patients with cancer that do not respond or develop resistance to KRAS G12C inhibitor treatment.

## Introduction

*RAS* is one of the most frequently mutated oncogenes. In fact, more than 20% of all human cancers are associated with mutations in one of the three RAS isoforms, KRAS, HRAS, or NRAS (1, 2). *KRAS* mutations are associated with a poor prognosis in general, notably in colorectal and pancreatic cancers. Mutations in *KRAS* are common in many solid tumors, most frequently occurring in 45% of colorectal, 35% of lung, and up to 90% of pancreatic cancers. In the United States alone, nearly 150,000 new cases of KRAS-mutated cancers are diagnosed each year across these three cancer types. More than half of all KRAS-driven cancers are caused by the three most common *KRAS* alleles, G12D, G12V, and G12C, which account for approximately 100,000 new cases in the United States (3).

The *KRAS* gene encodes a small GTPase that acts as a molecular switch controlling key signaling pathways, such as the MAPK (RAF/MEK/ERK) and PI3K (PI3K/AKT/mTOR) pathways, which are responsible for cell proliferation and survival. KRAS protein alternate between the GDP-bound (inactive) and GTP-bound (active) states. GTP-bound KRAS stimulates the activation of many downstream signaling pathways. A key feature of oncogenic KRAS is impaired GTP hydrolysis, which results in increased flux through downstream pathways (4).

KRAS has long been considered undruggable. Due to its high affinity for GTP/GDP and the lack of a clear binding pocket, efforts to directly target KRAS have largely failed (5, 6). However, a paradigm shift happened with the identification of a novel allosteric binding pocket under the switch II region of KRAS G12C protein that can be exploited for drug discovery. This led to the development of molecules that can covalently bind to G12C mutant KRAS at the cysteine 12 residue, thereby locking the protein in its inactive GDP-bound form that results in the inhibition of KRAS-dependent signaling, ultimately yielding antitumor activities (7–9). This opened a window of opportunity to selectively target KRAS G12C protein using effective mutant-specific small-molecule inhibitors, allowing KRAS to finally become druggable, albeit for a fraction of all KRAS-mutated tumors. Through this strategy several KRAS G12C inhibitors have been developed so far, including AMG510 (sotorasib) and MRTX849 (adagrasib). Both compounds have shown promising results in preclinical tumor models (10, 11) as well as in clinical trials (NCT03600883, NCT03785249). Sotorasib has recently become the first KRAS G12C inhibitor to be granted accelerated approval by FDA as a second-line treatment for patients with NSCLC carrying KRAS G12C mutation (12).

Although these KRAS G12C inhibitors have shown robust antitumor responses, but as targeted therapies, they are susceptible to the development of intrinsic or adaptive resistance, which can impede their prolonged therapeutic use (13, 14). There are already multiple indications that patients treated with these agents can develop drug resistance over time (15–18). This necessitates the need for combination approaches that can potentially sensitize tumors to KRAS inhibitors when cotargeted.

The nuclear export protein exportin 1 (XPO1) plays a vital role in maintaining cellular homeostasis by mediating the export of a number of protein cargoes, including the majority of tumor suppressor proteins (TSPs), from the nucleus to the cytosol (19). In many solid and hematologic malignancies, increased expression of XPO1 has been observed which reportedly correlated with poor prognosis (20–21). XPO1 overexpression enhances the export of TSPs to the cytosol, thereby preventing them from carrying out their normal function of cell growth regulation in the nucleus (22). Therefore, XPO1 inhibition that can cause sequestering of TSPs within the nucleus, has emerged as an appealing anticancer strategy (19).

Interestingly, it has been reported that KRAS-mutant NSCLC cells are dependent on XPO1-mediated nuclear export, rendering XPO1 a druggable vulnerability in KRAS-mutant lung cancer (23). Furthermore, the antitumor efficacy of XPO1 inhibitor selinexor against KRAS-mutant lung cancer patient-derived xenografts (PDX) was recently demonstrated (24). Moreover, XPO1 is linked to resistance to various standard-of-care chemotherapies and targeted therapies, which makes it a promising target for novel cancer therapies (25, 26). In fact, XPO1 inhibitor selinexor (in combination with dexamethasone alone and with bortezomib and dexamethasone) has been approved for patients with relapsed/refractory multiple myeloma (27) and as a monotherapy for patients with relapsed/refractory diffuse large B-cell lymphoma (28).

In this study, we report for the first time that KRAS G12C inhibitor–resistant cell lines show sensitivity toward selinexor, providing a rationale for testing XPO1 inhibitor in combination with KRAS G12C inhibitors as an effective combination therapy. Using KRAS G12C mutant *in vitro* and *in vivo* preclinical models, we demonstrate enhanced anticancer activity of selinexor and KRAS G12C inhibitor combinations.

## Materials and Methods

### Cell Lines, Drugs, and Reagents

MiaPaCa-2, NCI-H2122, NCI-H358, and Panc-1 cells were purchased from ATCC in 2012, 2013, 2021, and 2012, respectively. NCI “Rasless” mouse embryonic fibroblast (MEF) cell lines (KRAS 4B WT, G12C, G12D, G12V) were obtained from the NCI (Rockville, MD) in 2019. MiaPaCa-2, Panc-1, and all the MEF cell lines were maintained in DMEM (Thermo Fisher Scientific), while NCI-H2122 and NCI-H358 were maintained in RPMI1640 (Thermo Fisher Scientific), supplemented with 10% FBS, 100 U/mL penicillin, and 100 μg/mL streptomycin in a 5% CO_2_ atmosphere at 37°C. The cell lines have been tested and authenticated in a core facility of the Applied Genomics Technology Center at Wayne State University. The method used for testing was short tandem repeat (STR) profiling using the PowerPlex 16 System (Promega). *Mycoplasma* testing was routinely performed on the cell lines using PCR. All experiments were performed within 20 passages of the cell lines. MRTX1257 (ChemieTek), AMG510, MRTX849 (Selleck Chemicals LLC), and selinexor (Karyopharm Therapeutics) were dissolved in DMSO to make 10 mmol/L stock solutions. The drug control used for *in vitro* inhibitor experiments was cell culture media containing 0.1% DMSO.

### Generation of KRAS G12C Inhibitor (AMG510 and MRTX1257) Resistant Cell Lines

KRAS G12C–mutant pancreatic ductal adenocarcinoma (PDAC) cell line, MiaPaCa-2, was maintained in long-term cell culture exposed to incremental doses of AMG510 and MRTX1257 to develop drug resistance. MiaPaCa-2 cells, seeded at 60%–70% confluence in DMEM and 10% FBS, were maintained in fresh drug containing medium changed every 3 days. The cells were passaged once they reach −90% confluence. The starting doses of the drugs were half of the IC_50_. Doses were doubled after every fifth passage of cell culture. The maximum dose the cells were exposed to was four times the IC_50_. After about 3 months (20 passages) of continuous drug exposure, the resulting pool of cells were collected and named as MIA-AMG-R and MIA-MRT-R. These cells were then treated with varying concentrations of the respective inhibitors and MTT assay was performed. Drug resistance was estimated by comparing the fold change in IC_50_s of the drug-primed and the unexposed parental cells.

### Cell Viability Assay and Synergy Analysis

Cells were seeded in 96-well culture plates at a density of 3 × 10^3^ cells per well. The growth medium was removed after overnight incubation and replaced with 100 μL of fresh medium containing the drug at various concentrations serially diluted from stock solution using OT-2 liquid handling robot (Opentrons). After 72 hours of exposure to the drug, MTT (3-(4,5-dimethylthiazol-2-yl)-2,5-diphenyltetrazolium bromide) assay was performed according to the procedure described previously (29). Using the cell proliferation data (six replicates for each dose), IC_50_ values were calculated using the GraphPad Prism 4 software.

For the synergy analysis, cells were treated with three different concentrations of either MRTX1257/AMG510, or selinexor, or a combination of selinexor with MRTX1257/AMG510 at the corresponding doses for 72 hours (six replicates for each treatment). The drug proportion was kept constant across all the three dose combinations. Cell growth index was determined using MTT assay. The resulting cell growth data was used to generate isobolograms and calculate combination index (CI) values by the CalcuSyn software (Biosoft).

### Three-Dimensional Culture and Spheroid Formation Assay

MiaPaCa-2 and NCI-H2122 cells were trypsinized, collected as single-cell suspensions using cell strainer and resuspended in sphere formation medium which was composed of 1:1 DMEM and F-12 nutrient mix supplemented with B-27 and N-2 (Thermo Fisher Scientific). 1,000 cells were plated in each well of ultra-low attachment 6-well plates (Corning). Media was replenished every 3 days and spheroid growth was monitored. Spheroids growing in spheroid formation medium were exposed to either selinexor, or AMG510, or MRTX1257, or a combination of selinexor with either AMG510 or MRTX1257 twice a week for one week (three replicates for each treatment). At the end of the treatment, spheroids were counted under an inverted microscope and photographed.

### Colony Formation Assay

MiaPaCa-2 cells were seeded at a density of 500 cells per well in 6-well plates and exposed to single-agent or combination drug treatments for 72 hours. At the end of the treatment, drug containing media was removed and replaced with fresh media. The plates were incubated in the CO_2_ incubator for an additional ten days. After the incubation was over, media was removed from the wells of the plates and the colonies were fixed with methanol and stained with crystal violet for 15 minutes. The plates were then washed and dried before colonies were photographed. Colony number and sizes was later quantified by using NIH ImageJ 1.5Oi software.

### Preparation of Total Protein Lysates and Western Blot Analysis

1 × 10^6^ MiaPaCa-2 or NCI-H358 cells were grown in 10-cm petri dishes overnight. The following day, cells were treated with the drugs as single agents or combinations for 12 or 24 hours. For total protein extraction, cells were lysed in RIPA buffer and protein concentrations were measured using BCA protein assay (PIERCE). A total of 40 μg protein lysate from treated and untreated cells was resolved on 10% SDS-PAGE and transferred onto nitrocellulose membranes. The membranes were incubated with the following primary antibodies (Cell Signaling Technology) at 1:1,000 dilution in 3% non-fat dry milk: anti-phospho-P70 S6 Kinase (# 9204), anti-P70 S6 Kinase (# 9202), anti-phospho-ERK1/2 (# 4370), anti-ERK1/2 (# 9102), anti-cyclin B1 (# 12231), anti-CDK4 (# 12790). Anti-KRAS (# 517599; Santa Cruz Biotechnology), and anti-NF-κB p65 (# 06–418; Millipore Sigma) primary antibodies were also used at 1:1,000 dilution, while anti-β-actin (# sc-47778; Santa Cruz Biotechnology) and anti-GAPDH (# sc-47724; Santa Cruz Biotechnology) were used at a dilution of 1:3,000. Incubation with 1:2,000 diluted HRP-linked secondary antibodies (# 7074/7076; Cell Signaling Technology) in 3% non-fat dry milk was subsequently performed at room temperature for 1 hour. The signal was detected using the ECL chemiluminescence detection system (Thermo Fisher Scientific). Densitometric analysis of the data was performed using the ImageJ software (NIH, Bethesda, MD).

### Nuclear–Cytoplasmic Fractionation

Nuclear and cytoplasmic fractions of the cells were prepared using NE-PER Nuclear and Cytoplasmic Extraction Kit (Pierce Biotechnology), according to the manufacturer's instructions. Briefly, cells were detached using trypsin-EDTA and washed twice with PBS by centrifugation at 500 × *g* for 5 minutes. Ice-cold CER I was added to the cell pellet, mixed by vortexing, and CER II was added to the cells after 10 minutes of incubation. The tubes were vortexed, incubated for 1 minute, vortexed again, then centrifuged at maximum speed for 5 minutes at 4° C. The supernatant which contains the cytoplasmic extract was transferred to a new tube. The pellet which contains the nuclei was then washed with ice-cold PBS, then ice-cold NER was added. The tubes were incubated on ice for 40 minutes with vortexing every 10 minutes. The tubes were then centrifuged at maximum speed for 10 minutes, and the supernatant containing the nuclear extract was transferred to a new tube. Protein concentration of the fractions was measured, and the fractions were then used for subsequent Western blot analysis (as described above). Anti-Rb (# 9309; Cell Signaling Technology), anti-Lamin B1 (sc-377000; Santa Cruz Biotechnology) primary antibodies were used at 1:1,000 dilution, whereas anti-GAPDH (# sc-47724; Santa Cruz Biotechnology) was used at 1:2,000 dilution.

### KRAS G12C Cell–Derived Tumor Xenograft Study

*In vivo* studies were conducted under Wayne State University's Institutional Animal Care and Use Committee (IACUC) approved protocol in accordance with the approved guidelines. Experiments were approved by the institute's IACUC (Protocol # 18–12–0887). Post adaptation in our animal housing facility, 4–5 weeks old female ICR-SCID mice (Taconic Biosciences) were subcutaneously implanted with MiaPaCa-2 cells. 1 × 10^6^ cells suspended in 200 μL PBS were injected unilaterally into the left flank of donor mice using a BD 26Gx 5/8 1mL Sub-Q syringe. Once the tumors reached about 5%–10% of the donor mice body weight, the donor mice were euthanized, tumors were harvested, and fragments were subsequently implanted into recipient mice. Seven days posttransplantation, the recipient mice were randomly divided into four groups of 9 mice each and received either vehicle, or selinexor (15 mg/kg once a week), or AMG510 (100 mg/kg once daily), or their combination by oral gavage for 3 weeks. On completion of drug dosing, tumor tissue from control or treatment groups were used for RNA isolation and IHC analysis.

### RNA Isolation and mRNA Real-Time qRT-PCR

Total RNAs from mouse tumors were extracted and purified using the RNeasy Mini Kit and RNase-free DNase Set (QIAGEN) following the protocol provided by the manufacturer. The expression levels of *KRAS, XPO1, ERK2* and *BCL-2* in the mouse tumor tissues were analyzed by real-time qRT-PCR using High-Capacity cDNA Reverse Transcription Kit and SYBR Green Master Mixture from Applied Biosystems. The conditions and procedure for qRT-PCR have been described previously (29). Sequences of primers used are listed in Supplementary Table S1.

### Immunostaining

Paraffin sections of the MiaPaCa-2 derived tumors were processed and stained with H&E and antibodies in a core facility at the Department of Oncology, Wayne State University. The following antibodies were used for immunohistochemistry staining: anti-Ki67 (catalog no. M7240; Dako) and anti-KRAS (catalog no. 41–570–0; Fisher Scientific) at 1:100 dilution, and anti-cleaved caspase-3 (catalog no. 9664; Cell Signaling Technology) at 1:50 dilution.

### Statistical Analysis

The Student *t* test was used to compare statistically significant differences. Wherever suitable, the experiments were performed at least three times. The data were also subjected to unpaired two-tailed Student *t* test wherever appropriate, and *P* < 0.05 was considered statistically significant.

### Data Availability

The data generated in this study are available upon request from the corresponding author.

## Results

### Selinexor Induces Growth Inhibition in PDAC Cells Resistant to KRAS G12C Inhibitors

**FIGURE 1:**
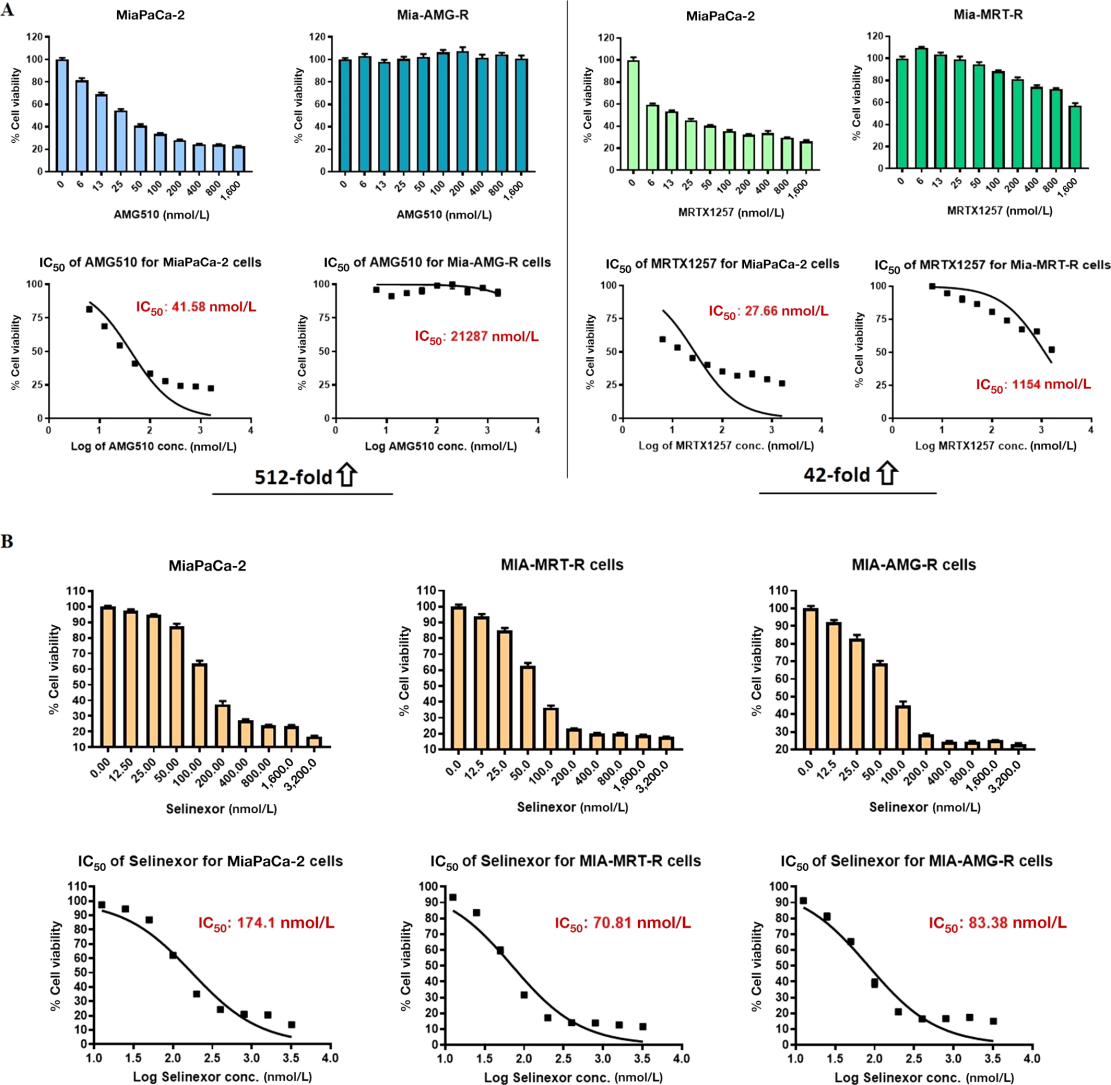
Selinexor induces growth inhibition in KRAS G12C inhibitor–resistant cancer cells. **A,** KRAS G12C–mutant MiaPaCa-2 cells exposed to incremental doses of AMG510 and MRTX1257 in long-term cell culture, eventually developed drug-resistance as shown by their unresponsiveness to drug treatment in MTT assay and several fold increase in the drug IC_50_ values compared with parental cells. **B,** AMG510- and MRTX1257-resistant MiaPaCa-2 cell lines show sensitivity toward selinexor induced growth inhibition. Parental as well as resistant cells were treated with selinexor for 72 hours and MTT assay was performed as described in Methods. All results are expressed as percentage of control ± SEM of six replicates.

KRAS G12C inhibitor–resistant cell lines were generated *in vitro* by continuous exposure of the KRAS G12C–mutant PDAC cell line MiaPaCa-2 to increasing doses of AMG510 and MRTX1257. To establish the development of drug-resistance, we compared the IC_50_ values of the drug-exposed cell lines with the unexposed parental line. We observed 512- and 42-fold increase in the IC_50s_ of AMG510-resistant (MIA-AMG-R) and MRTX1257-resistant (MIA-MRT-R) MiaPaCa-2 cells, respectively, confirming that the cells had developed resistance to the respective KRAS G12C inhibitors (Fig. 1A). Subsequently, both these drug-resistant cell lines (MIA-AMG-R and MIA-MRT-R) were treated with selinexor and were found to be sensitive to selinexor-induced cell growth inhibition (Fig. 1B). This establishes that the KRAS G12C inhibitor–resistant cancer cells can potentially respond to selinexor.

### Synthetic Lethal Interaction Between XPO1 and KRAS

A computational systems approach called SLant (Synthetic Lethal analysis via Network topology) has recently been used for the prediction of human synthetic lethal (SSL) interactions via identifying and exploiting conserved patterns in protein interaction network topology (30). We have obtained the experimentally validated synthetic lethal interactions of XPO1 using this approach from the Slorth database (http://slorth.biochem.sussex.ac.uk/welcome/index) and found interaction of XPO1 with KRAS to be synthetic lethal (Supplementary Fig. S1).

### Combining Selinexor with KRAS G12C Inhibitors Synergistically Suppresses the Proliferation of KRAS G12C–Mutant Cells

**FIGURE 2:**
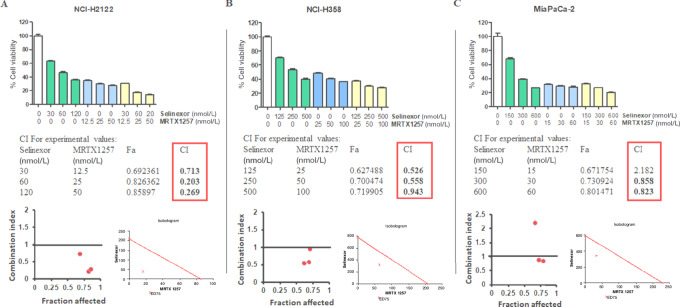
Selinexor and MRTX1257 show synergistic effects on the inhibition of cell proliferation *in vitro*. NCI-H2122 (**A**), NCI-H358 (**B**), and MiaPaCa-2 (**C**) cells were exposed to the indicated concentrations of either selinexor, MRTX1257, or a combination of both for 72 hours, and cell proliferation was evaluated by MTT assay as described in Materials and Methods. CalcuSyn software was employed to generate isobolograms and determine CI values from the resulting data. CI < 1 indicates synergistic effect of the drug combination at the corresponding doses. All results are expressed as percentage of control ± SEM of six replicates.

KRAS G12C–mutant NCI-H2122, NCI-H358 (NSCLC) and MiaPaCa-2 (PDAC) cells were subjected to *in vitro* MRTX1257 and selinexor treatments at different dose combinations. As shown in Fig. 2A and B, all three dose combinations tested demonstrated synergistic inhibition of NCI-H2122 and NCI-H358 cell proliferation (CI value < 1). For MiaPaCa-2 cells, synergistic effect (CI < 1) of the two drugs in suppressing cell growth was seen in at least two of the three combination doses tested (Fig. 2C). AMG510 and selinexor combinations have more of an additive effect on the growth inhibition of MiaPaCa-2 cells but showed mostly synergistic effects when tested on NCI-H2122 cells (Supplementary Table S2). These drug combinations were also tested on NCI “Rasless” MEFs carrying different *KRAS* mutations. Selinexor synergized with MRTX1257 at all dose combinations and with AMG510 at one of the higher dose combinations yielding suppressed growth of KRAS G12C mutant MEFs (Supplementary Table S2). As expected, the KRAS WT, KRAS G12D, and KRAS G12V MEF cell lines were refractory to any such growth inhibition (Supplementary Fig. S2). Similarly, the combination of selinexor with AMG510 showed no synergistic effects (CI > 1) when tested in KRAS G12D–mutant Panc-1 cells (Supplementary Fig. S3).

### Selinexor and KRAS G12C Inhibitor Combinations Effectively Disrupt the Formation of KRAS G12C–Mutant Cancer Cell–Derived Spheroids

**FIGURE 3:**
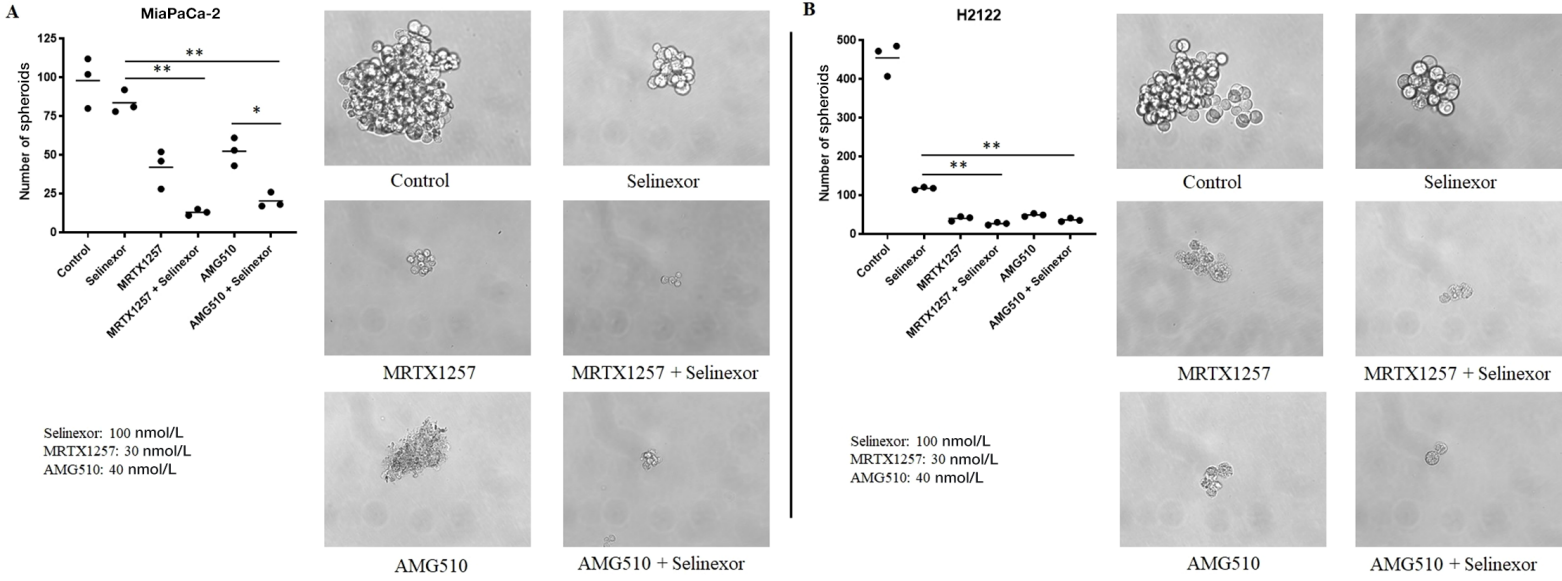
Selinexor in combination with MRTX1257 or AMG510 suppresses spheroid formation as well as significantly reduces the number of spheroids in 3D cultures of KRAS G12C–mutant cancer cells. MiaPaCa-2 (**A**) and NCI-H2122 (**B**) cells were seeded in ultra-low attachment plates and treated with indicated concentrations of the drugs either as single agents or in combination for a week. Each treatment was performed in triplicate. At the end of treatment, spheroids were counted under the microscope and images were captured at 40× magnification.

Sensitivity of cells in three-dimensional culture is considered to better predict *in vivo* efficacy, correlating well with drug response in xenograft models (31). Therefore, we performed a spheroid formation assay, where combined treatment of selinexor with either MRTX1257 or AMG510 resulted in enhanced disruption of spheroids derived from MiaPaCa-2 and NCI-H2122 cell lines (Fig. 3). Also, the total number of spheroids in the combination treated groups were significantly lower (*P* < 0.001) than that of the single-agent selinexor treated group. Only with the MiaPaCa-2–derived spheroids, significant reduction (*P* < 0.01) in spheroid numbers of the AMG510 and selinexor combination compared with AMG510 alone was observed. These results demonstrate the efficacy of selinexor and AMG510 or MRTX1257 combinations in three-dimensional (3D) cell growth models of KRAS G12C–mutant PDAC and NSCLC.

### Combination of Selinexor with KRAS G12C Inhibitors Reduces the Clonogenic Potential of KRAS G12C–Mutant Cancer Cells

**FIGURE 4:**
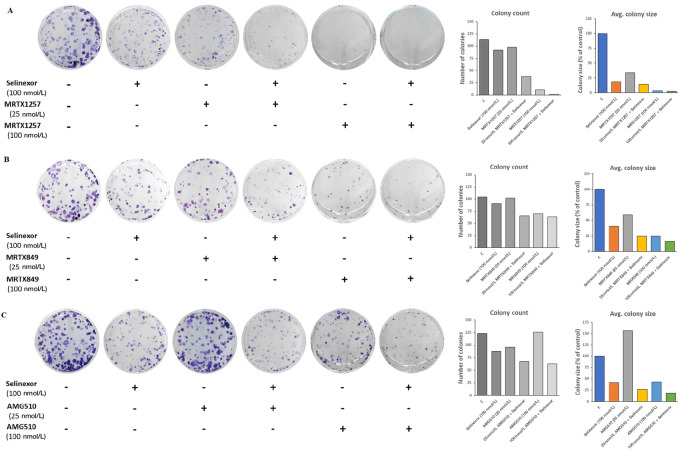
Combinations of selinexor with various KRAS G12C inhibitors inhibit the ability of KRAS G12C–mutant cancer cells to form colonies. MiaPaCa-2 cells were plated in 6-well plates (500 cells per well) and treated with combinations of selinexor with MRTX1257 (**A**), MRTX849 (**B**), and AMG510 (**C**) at the indicated concentrations for 72 hours and colony formation assay was performed as described in Methods. Images of crystal violet-stained colonies were captured and NIH ImageJ 1.5Oi software was used to measure the number and size of colonies. Data is representative of three independent experiments.

The combinations of selinexor with MRTX1257, MRTX849 or AMG510 were evaluated for their effects on the colony formation ability of MiaPaCa-2 cells. Results of a clonogenic assay clearly demonstrate that the combination treatments of selinexor with each of the KRAS G12C inhibitors resulted in substantial decline in colony numbers as well as reduced average size of colonies formed by MiaPaCa-2 cells (Fig. 4). Furthermore, this effect was more pronounced at the higher dose of KRAS G12C inhibitors tested (100 nmol/L). These findings further underscore the efficacy of this combination approach in targeting KRAS G12C mutant cancer cells *in vitro*.

### Selinexor and KRAS G12C Inhibitor Combinations Suppress Cell Growth Signaling and Prevent Cell-Cycle Progression

**FIGURE 5:**
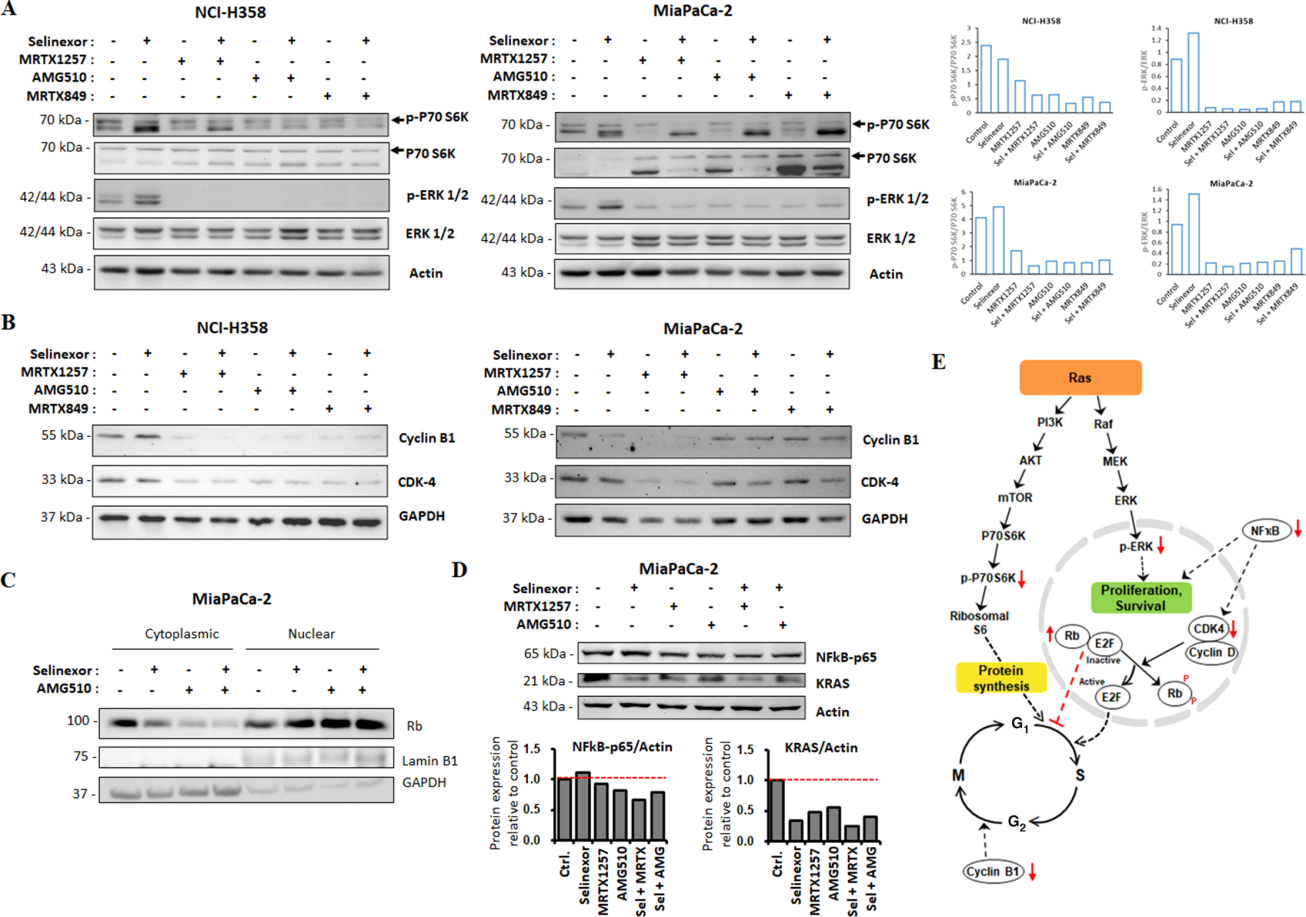
KRAS G12C inhibitors in combination with selinexor inhibit cell growth signaling and prevent cell-cycle progression. (**A**) Immunoblots showing suppression of P70 S6 Kinase and ERK activation in NCI-H358 and MiaPaCa-2 cells treated with MRTX1257, AMG510 and MRTX849 as single agents (300 nmol/L) and in combination with selinexor (100 nmol/L) for 24 hours. (**B**) Immunoblots showing the inhibition of cyclin B1 and CDK4 in NCI-H358 and MiaPaCa-2 cells treated with single-agent KRAS G12C inhibitors (300 nmol/L) or their combinations with selinexor (100 nmol/L) for 24 hours. (**C**) Immunoblot showing the expression of Rb in nuclear and cytosolic fractions of MiaPaCa-2 cells treated with AMG510 alone (300 nmol/L) or in combination with selinexor (100 nmol/L) for 24 hours. Lamin B1 and GAPDH were used as loading controls for the nuclear and cytosolic fractions, respectively. (**D**) Immunoblot showing reduced expression of KRAS and NF-κB in MiaPaCa-2 cells treated with selinexor (300 nmol/L) and MRTX1257 (90 nmol/L) or AMG510 (120 nmol/L) combinations for 12 hours. (**E**) Schematic of the proposed mechanisms by which the combination of KRAS G12C inhibitor and selinexor induces antineoplastic activity. This drug combination can block multiple nodes (represented with red arrows) of the cell proliferation and survival machinery. The quantitative analysis of mean pixel density of the blots was performed using NIH ImageJ 1.5Oi software.

The immunoblots in Fig. 5A clearly demonstrate that the combination of selinexor with KRAS G12C inhibitors downregulated P70S6K activation in NCI-H358 and MiaPaCa-2 cells. P70S6K is a mitogen-activated Ser/Thr protein kinase that is needed for cell growth and G_1_ cell-cycle progression. It is downstream of the PI3K/Akt cell survival pathway and is known to activate ribosomal S6, thereby promoting protein synthesis and cell growth. Hence, downregulation of S6K by the combination of selinexor and KRAS G12C inhibitors can result in suppression of cancer cell growth. Moreover, the combination was also able to sustain the inhibition of the ERK signaling pathway induced by the KRAS G12C inhibitors in both NCI-H358 and MiaPaCa-2 cells as indicated by the downregulation of ERK 1/2 phosphorylation (Fig. 5A). This effect on the direct inhibition of KRAS activated ERK signaling further contributes to the suppression of cancer cell proliferation.

Expression levels of cell-cycle markers such as CDK4 and cyclin B1 were also found to be reduced in both the cancer cell lines as a result of treatment with KRAS G12C inhibitors or their combinations with selinexor (Fig. 5B). Furthermore, we observed that the combination treatment augmented nuclear accumulation of tumor suppressor protein Rb (Fig. 5C). Acting as a tumor suppressor, Rb restricts cell division by preventing the progression from G_1_–S phase of cell cycle. CDK4-cyclin D complex phosphorylates Rb and releases this brake on cell-cycle progression. Evidently, it is likely that the combinations of selinexor and KRAS G12C inhibitors induce cell-cycle arrest in KRAS G12C–mutant cancer cells by downregulating cyclin B1 and CDK4 expression and upregulating the accumulation of TSP Rb in the nucleus. It can be envisaged that the reduced CDK4 levels led to reduction in Rb phosphorylation and consequently prevention of cell-cycle progression. This is possibly another mechanism through which this novel combination can mediate its anticancer effects.

### XPO1 and KRAS G12C Inhibitor Combination Downregulates KRAS and NF-κB Expression

As shown in Fig. 5D, the combination of selinexor with MRTX1257 or AMG510 potentiated the inhibition of KRAS expression in MiaPaCa-2 cells. NF-κB p65 protein expression was also markedly reduced in MiaPaCa-2 cells by selinexor and MRTX1257 combination compared with single agents. However, cells treated with selinexor and AMG510 combination showed only a slight decrease in the expression of NF-κB p65 in comparison to those treated with AMG510 alone. It was previously reported that the primary mechanism underlying XPO1 inhibitor sensitivity of KRAS-mutant lung cancer cell lines was intolerance to nuclear IκBα accumulation, with consequent inhibition of NF-κB signaling (28). Although our data show a minor increase in NF-κB p65 subunit expression in MiaPaCa-2 cells exposed to single-agent selinexor, the combination treatment nonetheless resulted in reduced NF-κB p65 expression, suggesting that the observed *in vitro* efficacy of the XPO1 inhibitor and KRAS G12C inhibitor combinations can be mechanistically attributed to the downregulation of NF-κB–driven cell survival signaling. On the basis of the molecular evidence obtained thus far, in Fig. 5E we have presented a schema of the possible mechanisms through which KRAS G12C inhibitor and selinexor combination can exert its effects on cancer cells. We believe that such a pleiotropic mode of action of this novel combination can render it more effective.

### AMG510 and Selinexor Combination is More Efficacious Than Single-Agent AMG510 in KRAS G12C–Mutant Cell-Derived Xenograft Model

**FIGURE 6:**
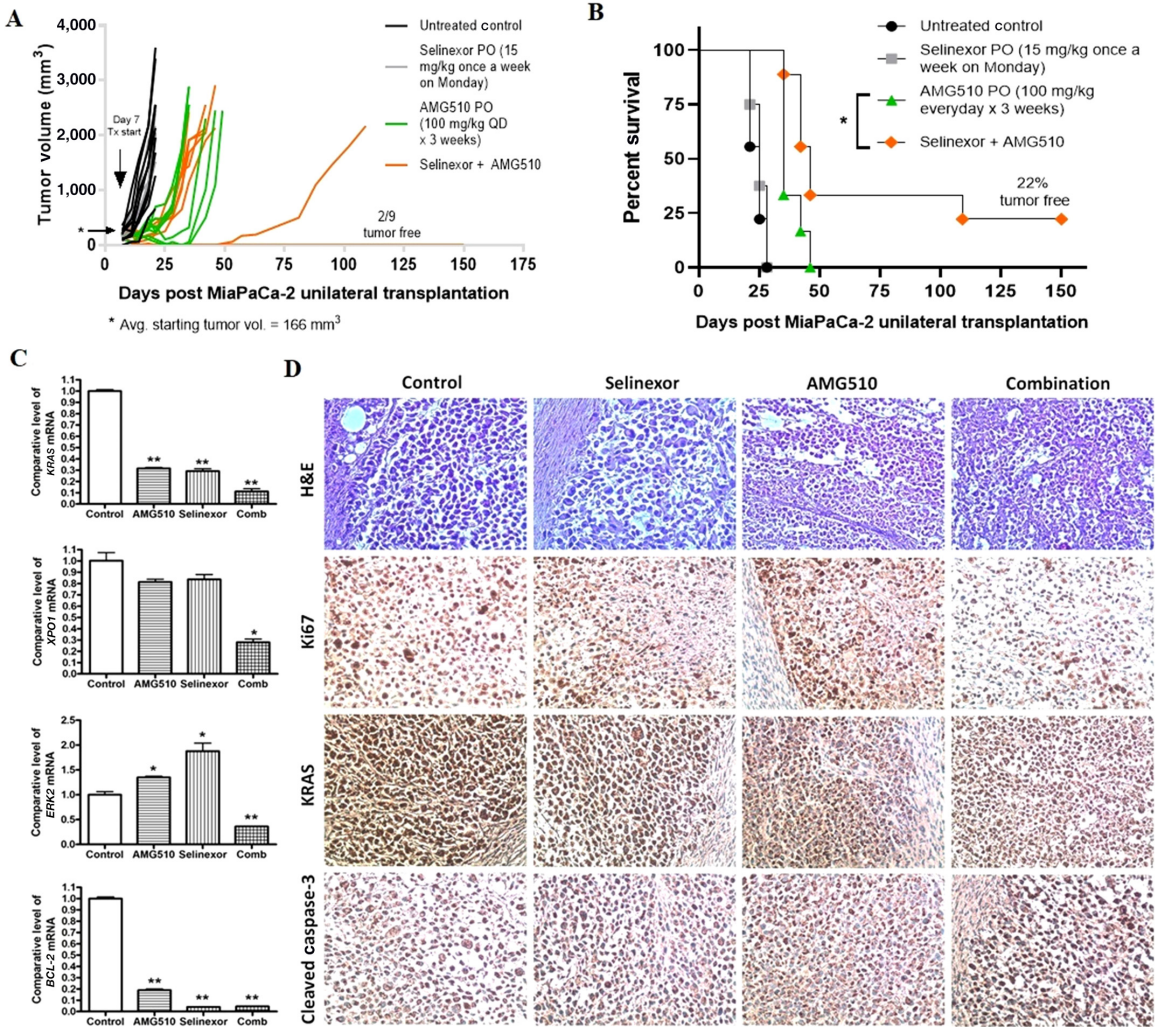
Preclinical antitumor efficacy of selinexor and KRAS G12C inhibitor combination in KRAS G12C CDX model. MiaPaCa-2 tumor xenografts were transplanted unilaterally in ICR-SCID mice, and the mice were randomly divided into four groups. Drug treatment was started one week after implanting xenografts when the average tumor volume reached 166 mm^3^. Selinexor was administered once a week at 15 mg/kg, while AMG510 was given daily at 100 mg/kg for 3 weeks. Tumor volume (**A**) and animal survival (**B**) were monitored up to 150 days posttransplantation. Residual tumor tissues from each group were used for measuring mRNA levels of *KRAS, XPO1, ERK2* and *BCL-2* (**C**), and performing IHC staining for Ki67, KRAS, and cleaved caspase-3 (**D**). PO, orally; QD, every day.

To evaluate the *in vivo* effect of AMG510 either as a single agent or in combination with selinexor, a subcutaneous xenograft model of MiaPaCa-2 cells was established in ICR-SCID mice. The tumor-bearing mice were orally treated with selinexor (15 mg/kg; once a week), AMG510 (100 mg/kg; daily) or the combination of AMG510 (100 mg/kg) and selinexor (15 mg/kg) for 3 weeks. Oral administration of AMG510 and selinexor combination showed greater tumor inhibition (Fig. 6A) as well as enhanced survival of mice harboring MiaPaCa-2 subcutaneous xenografts (Fig. 6B). Almost 22% of mice in the combination treatment group remained tumor free for as long as 150 days post tumor transplantation (Fig. 6B). The drug treatments, either single agents or combination, caused no significant change in body weights of mice during the course of the treatment (Supplementary Fig. S4). Also, we did not find any signs of either organ toxicity or metastatic spread while performing gross animal autopsy. In addition, the expression levels of *KRAS, XPO1, ERK2* and *BCL-2* mRNA were found to be significantly decreased in the residual tumor samples from the combination group (Fig. 6C). Further residual tumor profiling using IHC showed marked reduction in the proliferation marker, Ki67 and inhibition of KRAS in the combination group. In addition, the expression of proapoptotic marker, cleaved caspase-3 was high in the combination group (Fig. 6D). These results, taken together, demonstrate the safety and efficacy of AMG510 and selinexor combination *in vivo*.

## Discussion

In this article, we show synergy between KRAS G12C inhibitors and nuclear protein export inhibitor, for the first time. Our combination approach of cotargeting KRAS G12C and XPO1 resulted in enhanced growth suppression of KRAS G12C–mutant cells and cell-derived xenograft (CDX). This study brings forward a novel combination therapy for drug-resistant KRAS G12C–mutant tumors and provide preclinical rationale for the use of selinexor in a clinical setting to prevent or delay the development of resistance in patients receiving KRAS G12C inhibitor monotherapy.

Precision oncology has long sought to target KRAS oncoprotein directly. After decades of dismissing KRAS as untargetable, the development of inhibitors that can directly target KRAS G12C protein has rekindled hope. Researchers in the field have resumed their pursuit against KRAS with renewed vigor, especially after the early success of sotorasib and adagrasib in clinical trials (NCT03600883, NCT03785249). The recent FDA approval of sotorasib has surely widened the panorama of treatment for patients harboring KRAS G12C mutation. However, preliminary clinical data and prior experience with other targeted therapies, such as EGFR and BRAF inhibitors, suggest that there are still many hurdles to overcome. Just like other targeted therapies, KRAS G12C inhibitors are anticipated to have limited efficacy as monotherapies and resistance develops in most patients, necessitating the use of combination therapies (4). Hence, several combination approaches have been proposed, and some are currently undergoing clinical testing (11, 18, 32–34).

There is an increased realization for identifying KRAS-associated synthetic lethality and developing small-molecule inhibitors against such synthetic lethal targets. In a multi-genomic study, using 106 human NSCLC cell lines, Kim and colleagues (23) found that the nuclear transport machinery was selectively required for the survival of KRAS-mutant cells that carry a broad range of phenotypic variation. The study further demonstrated that targeting nuclear export protein XPO1 with selinexor resulted in a robust synthetic lethal interaction with oncogenic KRAS both *in vitro* and *in vivo*. The identification of the existence of synthetic lethality between XPO1 and KRAS using the Slorth database in this study further rationalizes the significance of cotargeting XPO1 and KRAS.

In another study, selinexor treatment was found to effectively reduce tumor growth in ten *KRAS*-mutant NSCLC PDXs irrespective of the type of *KRAS* mutation, indicating a general dependency of *KRAS*-mutant cancers on XPO1 (24). Moreover, *XPO1* was identified to be a dependency in at least 90% of cancer cell lines in a genome wide CRISPR/Cas9 screen performed on 808 cell lines (Cancer Dependency Map Project), putting it into the category of “common essential gene” (35). Also, multiple reports implicate *XPO1* to be a general vulnerability across several types of cancers (36–39). Inoue and colleagues have earlier shown that the administration of XPO1 inhibitor, followed by an ATR inhibitor, resulted in profound antitumor effects and prolonged survival in *TP53*-mutant colorectal cancer models (40). Similarly, in this study we have also achieved prolonged survival and antitumor effects with the use of XPO1 inhibitor in combination with KRAS G12C inhibitor in a KRAS G12C–mutant CDX model.

Because XPO1 is overexpressed in a number of cancers (20, 21), it appears that XPO1 mediated nuclear export may be harnessed by various cancers as a general mechanism of oncogenesis. Therefore, a combination therapy involving XPO1 and KRAS G12C inhibitors can be a viable option, especially considering that preclinical and clinical studies have already reported the emergence of resistant subpopulations of cancer cells in response to KRAS G12C inhibitor monotherapy (13–16). It can be speculated that these KRAS G12C inhibitor–resistant cancer cells would be eradicated by the use of XPO1 inhibitor as a combination partner. This proposition was validated when we generated two KRAS G12C inhibitor–resistant cancer cell lines from KRAS G12C–mutant parental cells (MiaPaCa-2) and found that both AMG510- and MRTX1257-resistant cell lines were indeed sensitive to the XPO1 inhibitor selinexor. Collectively, these findings imply that the inhibition of XPO1 activity could be a plausible therapeutic strategy for overcoming KRAS G12C resistance.

Our results demonstrate that the combinations of XPO1 inhibitor with KRAS G12C inhibitors can effectively inhibit the proliferation of KRAS G12C–mutant cancer cells in 2D and 3D cultures. These combinations have been further shown to remarkably suppress the clonogenic potential of KRAS G12C–mutant cancer cells. At the molecular level, these effects can be attributed to the ability of the combination to induce suppression of cell growth and survival signaling as well as preventing cell-cycle progression through downregulation of CDK4 and nuclear accumulation of tumor suppressor protein Rb. In an *in vivo* KRAS G12C CDX model of PDAC, increased efficacy of selinexor and sotorasib combination in suppressing tumor growth and enhancing survival has been observed. These results support the *in vitro* finding that selinexor treatment can sensitize KRAS G12C inhibitor–resistant cancer cells. Furthermore, this also suggests that combining selinexor with a KRAS G12C inhibitor sotorasib may have synergistic efficacy in patients with cancer that have developed resistance to therapy with sotorasib (or other KRAS G12C inhibitors). In this regard, we have planned a phase Ib/II study testing this combination in patients who have progressed on sotorasib. This novel combination therapy can potentially improve treatment outcomes in KRAS G12C–mutant cancers.

## Supplementary Material

Supplementary Figures 1-4, Tables 1-2 Supplementary Figure 1. XPO1-KRAS synthetic lethal interaction identified using the Slorth database. Supplementary Figure 2. KRAS WT, KRAS G12D and KRAS G12V MEF cell lines were refractory to growth inhibition by selinexor or MRTX1257 or their combinations. Supplementary Figure 3. KRAS G12D mutant PDAC cell line Panc-1 was refractory to growth inhibition by KRAS G12C inhibitor AMG510. Supplementary Figure 4. Body weights of mice xenografted with MiaPaCa2 tumors were measured during the treatment period. Supplementary Table 1. Sequences of primers used. Supplementary Table 2. Combination Index (CI) values at various dose combinations of the drugs tested on different KRAS G12C mutant cell lines.
