# Supplementary material for: Inhibitor of the Nuclear Transport Protein XPO1 Enhances the Anticancer Efficacy of KRAS G12C Inhibitors in Preclinical Models of KRAS G12C–Mutant Cancers
Source: Cancer Res Commun. 2022 May 10;2(5):342–52. doi: 10.1158/2767-9764.CRC-21-0176 (PMC9105196; doi:10.1158/2767-9764.CRC-21-0176)
Supplement: Supplementary Figures 1-4, Tables 1-2 — Supplementary Figure 1. XPO1-KRAS synthetic lethal interaction identified using the Slorth database. Supplementary Figure 2. KRAS WT, KRAS G12D and KRAS G12V MEF cell lines were refractory to growth inhibition by selinexor or MRTX1257 or their combinations. Supplementary Figure 3. KRAS G12D mutant PDAC cell line Panc-1 was refractory to growth inhibition by KRAS G12C inhibitor AMG510. Supplementary Figure 4. Body weights of mice xenografted with MiaPaCa2 tumors were measured during the treatment period. Supplementary Table 1. Sequences of primers used. Supplementary Table 2. Combination Index (CI) values at various dose combinations of the drugs tested on different KRAS G12C mutant cell lines. [file crc-21-0176-s01.pdf]

Supplementary Figure 1.

12/16/21, 11:50 PM Synthetic lethal interaction index - Slorth

Gene: xpo1 Organism: H. sapiens Type: SSL Quality: ? (/welcome/help#quality) Experimentally Validated Search

More / less options

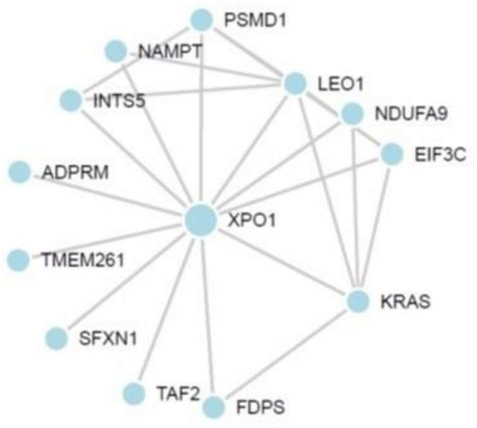

Interactions

Gene ID A	Gene name A	Gene ID B	Gene name B	Organism	Type	Source	Score ? (/welcome/help#score)	
ENSG00000139180	NDUFA9 (/genes/649)	ENSG00000082898	XPO1 (/genes/1061)	H. sapiens	SSL	BioGRID	1.0	View (/interactions/3334)
ENSG00000166477	LEO1 (/genes/540)	ENSG00000082898	XPO1 (/genes/1061)	H. sapiens	SSL	BioGRID	1.0	View (/interactions/2858)
ENSG00000173692	PSMD1 (/genes/754)	ENSG00000082898	XPO1 (/genes/1061)	H. sapiens	SSL	BioGRID	1.0	View (/interactions/3644)
ENSG00000164466	SFXN1 (/genes/830)	ENSG00000082898	XPO1 (/genes/1061)	H. sapiens	SSL	BioGRID	1.0	View (/interactions/3908)
ENSG00000170222	ADPRM (/genes/30)	ENSG00000082898	XPO1 (/genes/1061)	H. sapiens	SSL	BioGRID	1.0	View (/interactions/82)
ENSG00000184110	EIF3C (/genes/322)	ENSG00000082898	XPO1 (/genes/1061)	H. sapiens	SSL	BioGRID	1.0	View (/interactions/1686)
ENSG00000064313	TAF2 (/genes/867)	ENSG00000082898	XPO1 (/genes/1061)	H. sapiens	SSL	BioGRID	1.0	View (/interactions/3999)
ENSG00000185085	INTS5 (/genes/492)	ENSG00000082898	XPO1 (/genes/1061)	H. sapiens	SSL	BioGRID	1.0	View (/interactions/2488)
ENSG00000137038	TMEM261 (/genes/881)	ENSG00000082898	XPO1 (/genes/1061)	H. sapiens	SSL	BioGRID	1.0	View (/interactions/4031)
ENSG00000133703	KRAS (/genes/7)	ENSG00000082898	XPO1 (/genes/1061)	H. sapiens	SSL	BioGRID	1.0	View (/interactions/2767)
ENSG00000105835	NAMPT (/genes/642)	ENSG00000082898	XPO1 (/genes/1061)	H. sapiens	SSL	BioGRID	1.0	View (/interactions/3305)
ENSG00000160752	FDPS (/genes/383)	ENSG00000082898	XPO1 (/genes/1061)	H. sapiens	SSL	BioGRID	1.0	View (/interactions/2038)

sloth.biochem.sussex.ac.uk/interactions?utf8=✓&gene=xpo1&organism=H.+sapiens&interaction_type=SSL&score_filter=1&commit=Search&disease... 1/2

XPO1-KRAS synthetic lethal interaction identified using the Slorth database (http://slorth.biochem.sussex.ac.uk/welcome/index)

Supplementary Figure 2.

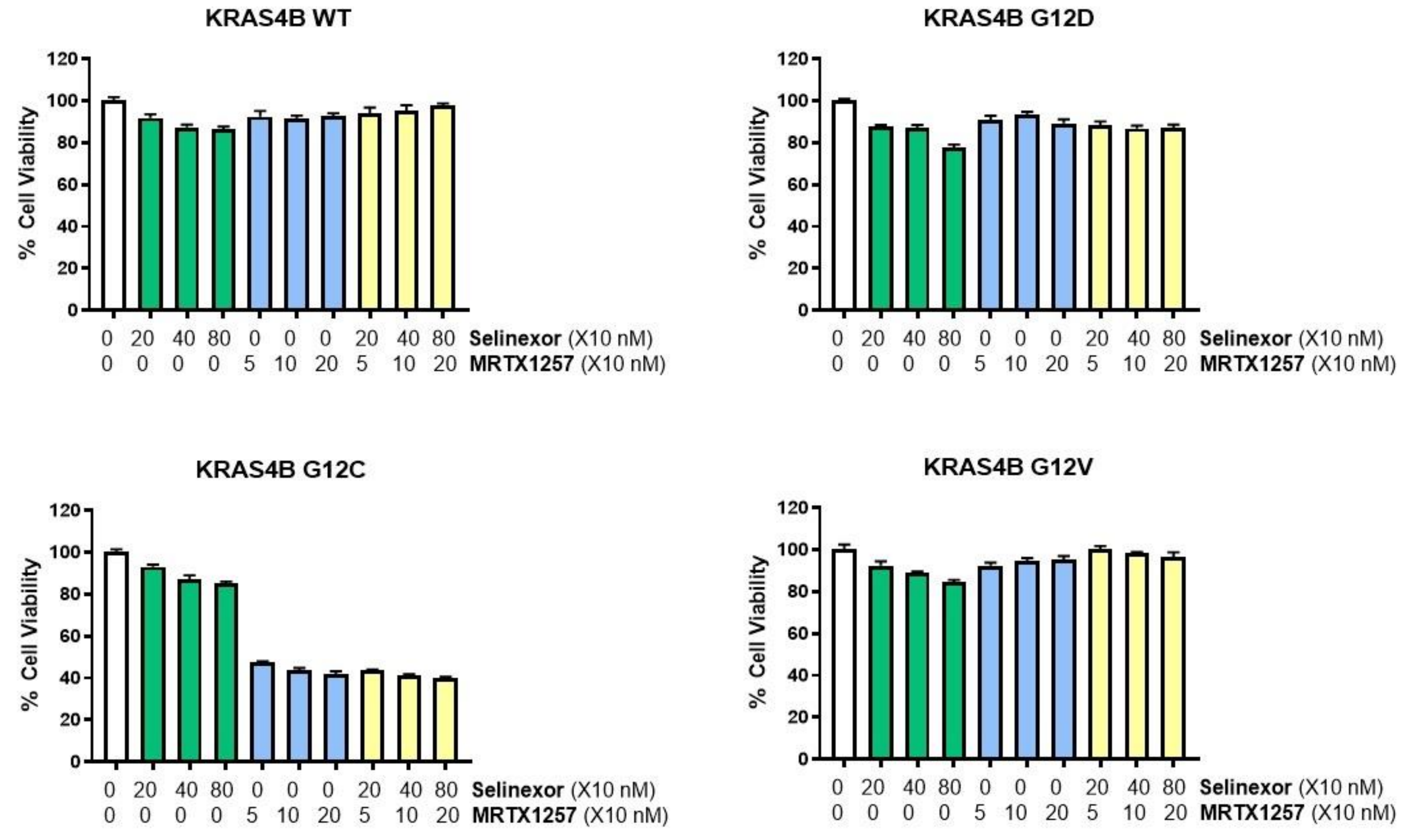

KRAS WT, KRAS G12D and KRAS G12V MEF cell lines were refractory to growth inhibition by selinexor or MRTX1257 or their combinations. However, only KRAS4B G12C cells responded to KRAS G12C inhibitor MRTX1257.

Supplementary Figure 3.

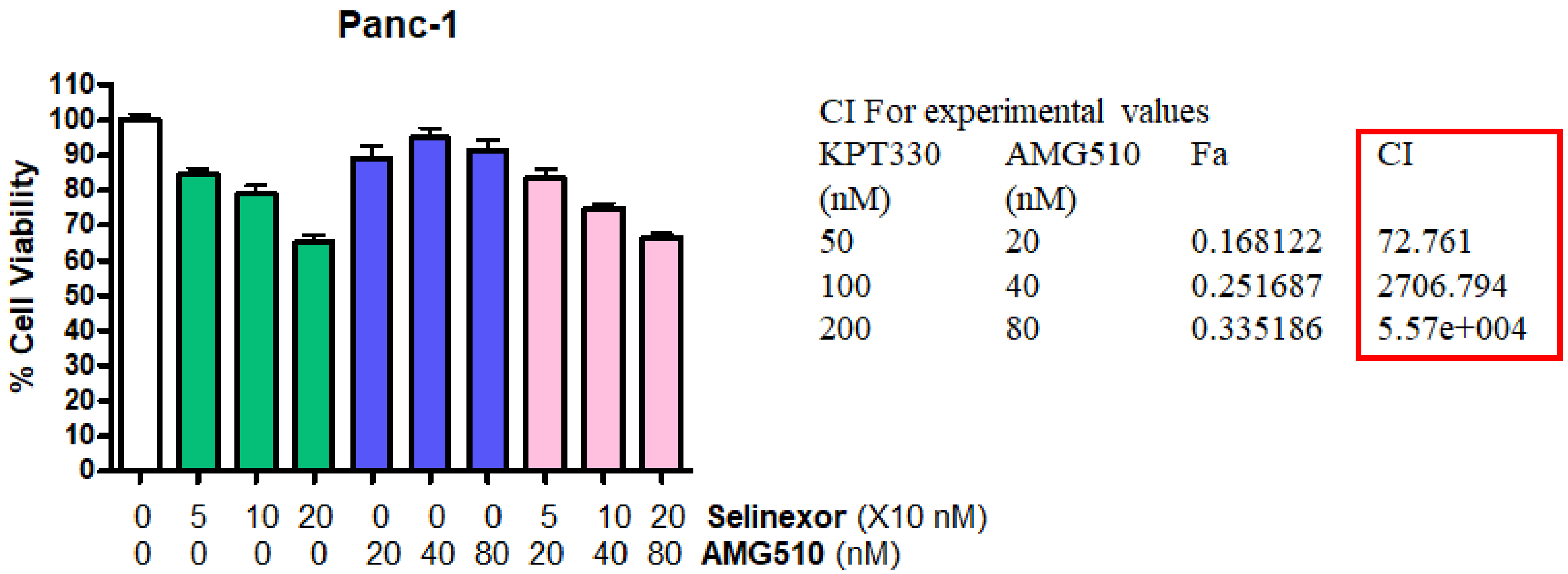

CI For experimental values

KPT330 (nM)	AMG510 (nM)	Fa	CI
50	20	0.168122	72.761
100	40	0.251687	2706.794
200	80	0.335186	5.57e+004

KRAS G12D mutant PDAC cell line Panc-1 was refractory to growth inhibition by KRAS G12C inhibitor AMG510. Treatment of Panc-1 cells with combinations of selinexor and AMG510, did not show any synergistic effect (CI > 1).

Supplementary Figure 4.

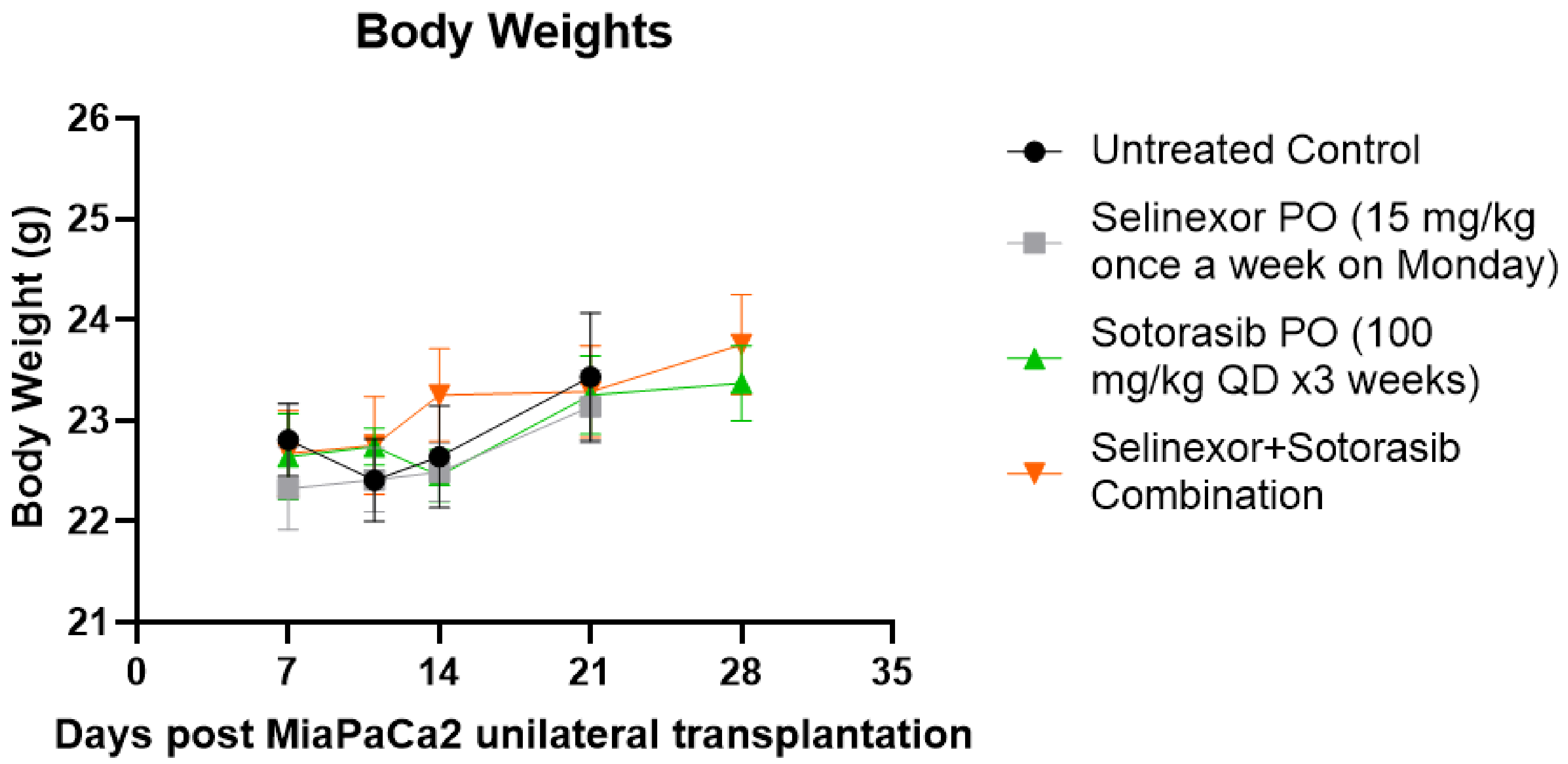

Body weights of mice xenografted with MiaPaCa2 tumors were measured during the treatment period. Neither of the drugs, either as single agent or in combination, induced any loss of body weight.

Supplementary Table 1. Sequences of primers used

Primers	Sequences	
KRAS	*Forward*	GTGCAAGAGAGCTGAGGGAG
	Reverse	ATGCTGGTGGGACAGAAGTG
XPO1	*Forward*	GGAAAACTGTGAAACCCACCTT
	Reverse	GCTGCATGGTCTGCTAACAT
Erk2	*Forward*	ATTACGACCCGAGTGACGAG
	Reverse	GATGTCTGAGCACGTCCAGT
Bcl-2	*Forward*	TGAACTGGGGGAGGATTGTG
	Reverse	CGTACAGTTCCACAAAGGCA
β-actin	*Forward*	GCACAGAGCCTCGCCTT
	Reverse	TCATCATCCATGGTGAGCTG
18S	*Forward*	GCAATTATTCCCCATGAACG
	Reverse	GGCCTCACTAAACCATCCAA

Supplementary Table 2. Combination Index (CI) values at various dose combinations of the drugs tested on different KRAS G12C mutant cell lines (MiaPaCa-2, NCI-H2122 and KRAS4B G12C)

MiaPaCa-2		
**Selinexor (nM)**	**AMG510 (nM)**	**CI**
50	20	0.927
100	40	1.162
200	80	1.554
NCI-H2122		
**Selinexor (nM)**	**AMG510 (nM)**	**CI**
150	50	0.986
300	100	0.931
600	200	0.781
KRAS4B G12C		
**Selinexor (nM)**	**MRTX1257 (nM)**	**CI**
200	50	0.41
400	100	0.451
800	200	0.627
KRAS4B G12C		
**Selinexor (nM)**	**AMG510 (nM)**	**CI**
800	800	0.103
